# Effects of woody forages on biodiversity and bioactivity of aerobic culturable gut bacteria of tilapia (*Oreochromis niloticus*)

**DOI:** 10.1371/journal.pone.0235560

**Published:** 2020-07-02

**Authors:** Feng Wu, Biao Chen, Sha Liu, Xiongjian Xia, Liuling Gao, Xiaoyong Zhang, Qing Pan

**Affiliations:** 1 Joint Laboratory of Guangdong Province and Hong Kong Region on Marine Bioresource Conservation and Exploitation, College of Marine Sciences, South China Agricultural University, Guangzhou, China; 2 Guangdong Laboratory for Lingnan Modern Agriculture, Guangzhou, China; Tallinn University of Technology, ESTONIA

## Abstract

The present study investigated the effects of four woody forages (*Moringa oleifera Lam* (MOL), fermented MOL, *Folium mori* (FM) and fermented FM) on biodiversity and bioactivity of aerobic culturable gut bacteria of tilapia (*Oreochromis niloticus*) by a traditional culture-dependent method. A total of 133 aerobic culturable isolates were recovered and identified from the gut of tilapia, belonging to 35 species of 12 genera in three bacterial phyla (Firmicutes, Actinobacteria and Proteobacteria). Among them, 6 bacterial isolates of *Bacillus baekryungensis*, *Bacillus marisflavi*, *Bacillus pumilus*, *Bacillus methylotrophicus*, *Proteus mirabilis* and *Pseudomonas taiwanensis* were isolated from all the five experimental groups. The Bray-Curtis analysis showed that the bacterial communities among the five groups displayed obvious differences. In addition, this result of bioactivity showed that approximate 43% of the aerobic culturable gut bacteria of tilapia displayed a distinct anti-bacterial activity against at least one of four fish pathogens *Streptococcus agalactiae*, *Streptococcus iniae*, *Micrococcus luteus* and *Vibrio parahemolyticus*. Furthermore, *Bacillus amyloliquefaciens* and *Streptomyces rutgersensis* displayed strong activity against all four indicator bacteria. These results contribute to our understanding of the intestinal bacterial diversity of tilapia when fed with woody forages and how certain antimicrobial bacteria flourished under such diets. This can aid in the further exploitation of new diets and probiotic sources in aquaculture.

## Introduction

Tilapia is popular among consumers in most regions and countries, with a global consumption and output that continue to increase [1, 2]. However, because of the large amount of tilapia cultured, even though the demand of fish meal in tilapia aquaculture is very low, the consumption of fish meal still accounts for a large proportion of the overall cost [3]. Although the fish meal is the best sources of quality proteins used in aquaculture diets, its application is limited because it is relatively costly [4, 5] and it can lead to water pollution [6]. Therefore, finding some new protein sources used in aquaculture feed has become a focus of aquaculture research [7].

Plant-derived forage has advantages of being obtained from a wide variety of sources and being low cost. With respect to animal-derived feed, plant-derived feed broadens aquaculture feed resources and reduces feed costs [8]. Moreover, some plant-derived forages with high nutritional value and that are rich in amino acids have been widely developed and utilized. Recently, cotton meal [9], soybean meal and peanut meal [10, 11] have been widely used in aquaculture feed and are very valuable.

Some woody plants with nutritional value and low price are ideal new feed for aquatic animals [12]. Woody materials like *Moringa oleifera* Lam (MOL), the dry leaves of which contain 30.3% crude protein [13], and *Folium mori* (FM) and *Broussonetia papyrifera* fruits containing some antioxidant substances [14, 15]. Furthermore, the woody plants have great resource advantages, and their utilization needs to be improved in China. In previous studies, Rugang Han [16] used MOL leaf meal as aquatic feed, while Kaviraj et al. [17] used fermented FM to feed the Indian major carp *Labeo rohita*. On the other hand, Lim et al. [18] and Zaineldin et al. [19] have shown that the rational use of microbial fermentation technology can produce fermented feeds that can improve feed digestibility, feed intake and immunity of aquatic animals. These recent studies have indicated that the application of woody forages, even after their fermentation, in aquatic animal feed has broad application.

In recent years, studies have shown that woody forage has a great impact on fish intestinal microbes [20]. After feeding the golden pompano *Trachinotus ovatus* woody forage or reference feed, the intestinal bacterial communities of the two groups was different at the phylum level. The most abundant phyla in fish fed the reference feed were Fusobacteria (30.2%), Firmicutes (29.7%), Proteobacteria (27.3%) and Bacteroidetes (9.5%), whereas Proteobacteria (39.1, 23.8, 16.4 and 46.1%), Firmicutes (17.5, 46.7, 55.9 and 18.2%), Fusobacteria (26.7, 14.8, 17.1 and 14.9%) and Bacteroidetes (8.2, 10.7, 9.0 and 7.7%) were the dominant phyla in the fish fed FM, MOL, *Neolamarckia cadamba* and *Broussonetia papyrifera*, respectively. The observed differences might be due to the presence of bacteria in the intestinal wall, mucosa and contents of tilapia [21]. Insights into the microbial diversity present in the intestines of fish may increase our understanding of the metabolic potential of intestinal microorganisms, the roles of some feed additives and the host health [22, 23]. In addition, because fish are exposed to a very high microbial load in the aquatic environments, this close contact with the surrounding water likely affects early gut colonization [24]. Water appears to affect the fish gut microbiota from the mouth onwards [25], but feed has important effects on the fish gut microbiome at a later development stage [26, 27]. Intestinal microorganisms play important roles in nutrient metabolism secreting number of digestive enzymes, and growth factors that promote the metabolic activities of animals [28]. Few studies have investigated the effect of woody feed on the aerobic culturable intestinal bacteria of aquatic animals.

Thus, the first objective of the present study was to explore the effects of four woody forages, including MOL leaf meal, FM meal, fermented MOL leaf meal and fermented FM meal, on the aerobic culturable intestinal bacteria present in tilapia to gain a theoretical basis for the application of the woody forages in the feed of tilapia and other types of fish.

The increased interest during the last decade on bacteria in the gut of fish is related to the fact that some bacteria often produce bacteriocins and other chemical compounds that may inhibit colonization of pathogenic bacteria in the gut. Therefore, the second aim of the present study was whether it is possible to find bacteria that inhibit pathogens in vitro through culturable methods.

## Materials and methods

The study was conducted under the guidelines of the Animal Ethics Committee at the South China Agricultural University, China (approval ID: SYXK-2019-0136).

### Woody forage

The ingredients of the reference diet group (the control group) in this study are shown in Table 1. The reference (Ref) diet was prepared according to the protocol of Pan et al [29]. The nutrient compositions of the five diets were controlled similarly, containing 5.00% moisture, 32.00% of crude protein, 5.20% of crude lipid, 10.40% crude ash and 1.15% phosphorus. In the process of feed fermentation, the activated strains are first expanded and mixed with fermentation materials (leaf powder, starch and other additives), and then the mixed fermentation materials and strains are fully mixed and placed in fermentation bags and placed in an artificial climate room for fermentation 9 days. And the mixed all raw materials was extruded into pellet diet with 2 mm in diameter by twin screw extruders (VALVA60-III, Guangzhou, China), placed in an air-conditioned room and dried in the air, then sieved and placed in a sealed bag at -20°C keep in the refrigerator for use. For non-fermented feed, the fermentation process is omitted directly.

**Table 1:** Feed formulation and proximate composition of the five test feeds (%).

	Cont.	MOL	FMOL	FM	FFM
soybean meal	45.00	36.50	36.50	39.00	39.00
rapeseed meal	10.00	14.50	14.50	12.00	12.00
wheat flour	34.99	12.89	7.89	10.89	8.39
starch			5.00		2.50
tapioca flour		5.00	5.00	3.00	3.00
wheat gluten				4.00	4.00
freshwater fish meal	3.00	3.00	3.00	3.00	3.00
fresh water fish oil	2.00	2.00	2.00	2.00	2.00
soybean lecithin	1.00	1.00	1.00	1.00	1.00
mono-calcium phosphate	2.00	2.00	2.00	2.00	2.00
feed additive premix	2.01	3.11	3.11	3.11	3.11
MOL meal		20			
FMOL meal			20		
FM meal				20	
FFM meal					20
Analyzed proximate composition (%)					
Moisture	5.10	5.40	4.63	6.44	4.52
Crude protein	32.60	32.90	32.59	31.96	32.91
Crude lipid	5.12	5.37	5.25	5.05	5.38
Crude ash	10.17	10.32	10.77	10.26	10.61
Phosphorus	1.22	1.12	1.09	1.14	1.11

The five groups respectively are control group (Cont.), *Moringa oleifera* Lam group (MOL), fermented *Moringa oleifera* Lam group (FMOL), *Folium mori* leaf meal group (FM) and fermented *Folium mori* leaf meal group (FFM).

### Experimental fish and feeding management

Tilapia were purchased from the National Tilapia Breeding Farm of Guangdong Province, China, and their initial weight was 70.0 ± 4.0 g. The fish were acclimated in large net cages in a pond for one week, after which 1200 fish were randomly stocked into 20 net cages, with 60 fish per cage. Each forage was assigned in 4 replicate cages. The fish were fed five different diets two times daily for 8 weeks [30]. During the period, we will check the pH value, temperature, dissolved oxygen content and ammonia nitrogen content of the net cage every 6 hours and keep these values in the following range: the pH value and temperature were 7.6–7.8 and 25–32°C, respectively, the dissolved oxygen content was greater than 4 mg/l and the ammonia nitrogen content was less than 0.1 mg/l.

### Sample collection

At the end of the feeding trial, feed was withheld from fish for 24 h. Additionally, three fish from each treatment group were anesthetized by tricaine methane sulphonate (MS 222, 100 ppm) to collect the gut samples [31]. Three fish from each group were randomly selected for aerobic culturable bacterial isolation. Under aseptic conditions, the gut of each fish without intestinal contents were collected in frozen tubes and stored under -80°C.

### Bacterial isolation and comparison

Approximately 100 mg of the thawed intestinal sample was thoroughly ground with1 ml of sterile water. After being diluted 10-fold, 0.1 ml of the bacterial fluid was spread onto prepared MRS [32] (enzymatic digest of casein 10 g/l, meat extract 10 g/l, yeast extract 4 g/l, triammonium citrate 2 g/l, sodium acetate 5 g/l, MgSO4·7H2O 0.2 g/l, glucose 20 g/l, MnSO4·4H2O 0.05 g/l, tween-80 1.08 g/l, dipotassium hydrogen phosphate 2 g/l), LB [33] (peptone 10 g/l, yeast extract 5 g/l, sodium chloride 5 g/l) and PIYCA (peptone 5 g/l, iron phosphate 0.1 g/l, yeast extract 1 g/l, casein 5 g/l) agar plates (at least three plates per medium). The plates were cultured for 2 days at 30°C to observe the bacterial morphology. According to the morphology, some isolates could be distinguished. Subsequently, different bacterial isolates were selected and transferred to new LB agar plates that were incubated at 30°C to pure cultures [34].

### Molecular characterization and identification of the bacterial isolates

Take a single colony and add 5mL of DNA extraction buffer (100mmol / L Tris-HCl, 100mmol / L EDTA-Na2, 1mol / L NaCl, 1% CTAB pH 8.0), then add 10% SDS 800μL and mix and incubate at 37°C for 1h. During this period, the mixture was continuously mixed several times, and an equal volume of saturated phenol: chloroform: isoamyl alcohol (25: 24: 1) was added to the centrifuge tube and centrifuged at 12 000 r / min for 10 minutes and extracted twice, and then chloroform: isoamyl alcohol (24: 1) Centrifuge at 12 000r / min for 10min and extract once. Take the supernatant in a new centrifuge tube and add 0.6 volumes of isopropanol to precipitate DNA for 1h, then centrifuge at 12 000r / min for 10min and discard the supernatant. The DNA pellet was dissolved in 0.1 mL TE buffer (10 mmol / L Tris-HCl, 1 mmol / L EDTA, pH 8.0 with RNase enzyme), and then heated at 100°C for 30 min [35, 36]. The mixture was then centrifuged at 10,000 r/m for 10 min at 4°C to obtain the template DNA. The bacterial universal primers 27F (5′-AGAGTTTGATCCTGGCTCAG-3′) and 1492R (5′-GGTTACCTTGTTACGACTT-3′) were used to PCR amplify the 16S rDNA sequence (Eppendorf AG 22331 Hamburg 6331). PCR amplifications were performed with an initial denaturation temperature of 95°C for 5 min; followed by 30 cycles of 95°C for 60 s, 55°C for 60 s (primer annealing) and 72°C for 90 s (primer extension), with a final primer extension step at 72°C for 10 min [37]. Sequencing of the 16S rDNA of the selected isolates was performed by BIOTREE (China). Sequences that were obtained using National Center for Biotechnology Information (NCBI) databases were compared with Basic Local Alignment Search Tool (BLAST) searches [38]. When the relatively best matches were from the same species and were 95% similar to the query sequence, this species name was assigned to the selected isolate [39].

### Data analysis

The Bray-Curtis analysis [40] was performed using SPSS and the diversity factor of the Bray-Curtis analysis was estimated, basing on the presence/absence matrix of intestinal bacteria obtained from fish in the five groups [41, 42].

### Determination of bioactivity (antibacterial activity)

The bioactivity (antibacterial activity) of 35 bacterial representative isolates recovered in this study was determined by a double-layer technique [43, 44]. The selected bacterial representative isolates were grown on LB media that was allowed to grow for 1–2 days depending upon the growth rate of the various isolates. The agar blocks containing the cells were the excised and placed on the assay plates spread with 4 indicator microorganisms (four fish pathogens) including *Streptococcus agalactiae* (SA), *Streptococcus iniae* (SI), *Micrococcus luteus* (ML), *Vibrio parahemolyticus* (VP) [45–48]. The assay plates with agar blocks and indicator microorganisms were cultivated at 37°C for 18–36 hours. The antimicrobial activity was expressed as the diameter of the growth inhibition zone (in milliliter). Each test was performed three times. All the indicator microorganisms were from the Joint Laboratory of Guangdong Province and Hong Kong Region on Marine Bioresource Conservation and Exploitation, China. After the initial evaluation for anti-bacterial activity, the minimum inhibitory concentrations (MICs) of the active extracts were further determined by a microtitre plate assay [49]. First, connect fresh *Bacillus altitudinis*, *Bacillus amyloliquefaciens*, *Bacillus pumilus* to the sterilized 200 mL LB liquid medium (tryptone 10 g/l, yeast extract 5 g/l, NaCl 10 g/l and sea salt 30 g/l) at 1% inoculation volume, and incubate at 30°C, 180 r / min for 18h, then ferment the broth at 10000 r / min. After centrifugation for 5 min, the supernatant was filtered with a 0.22 μm microporous filter membrane to obtain a sterile filtrate. Sterile filtrate and n-butanol were subjected to ultrasonic extraction at a ratio of 1: 7 for 0.5h. The extract layer was collected and concentrated under reduced pressure using a rotary evaporator at 45°C. The resulting crude extract was dissolved in 1 mL of methanol and stored at 4°C until use [50]. And, *Staphylococcus sp*., *Streptomyces rutgersensis* strain was activated, cultured at 28°C, and seed liquid was prepared. Choose a 250 mL Erlenmeyer flask with the same specifications and fill it with 50 mL RA medium (soluble starch 20 g (boiling and dissolving), glucose 10g, maltose 10g, malt extract powder 10g, corn flour 5 g, CaCO_3_ 2 g, pH 7.5), The seed solution was inoculated into three kinds of sterilized fermentation medium, and cultured with shaking at 200 r / min at 28°C for 7 days. The fermentation supernatant was mixed with an equal volume of butanone for ultrasonic extraction, the butanone extract layer was collected, concentrated under reduced pressure at 45°C on a rotary evaporator, and the crude extract was dissolved in 1 mL of methanol, and stored at 4°C until use [51]. Finally, the overnight culture of bacteria was adjusted to an optical density of 0.6 at 600 nm in the corresponding medium. Then, transfer the active extracts of different concentrations (50, 25, 12.5, 6.2, 3.1, and 1.6 μg / ml) to the wells of a 96-well polystyrene microtiter plate (100 μl per well) at 30°C Incubate for 24h. The MIC was recorded as the lowest concentration of active extract at which no bacterial growth was observed. Each extract was performed in triplicate with the positive control (*streptomycin*).

## Results

### BLAST searches and analysis of 16S rDNA gene sequences

The data obtained from the 16S rDNA sequences of 38 representative bacterial isolates were deposited in GenBank. The numbers are MK281515-MK281549.

**Fig 1:**
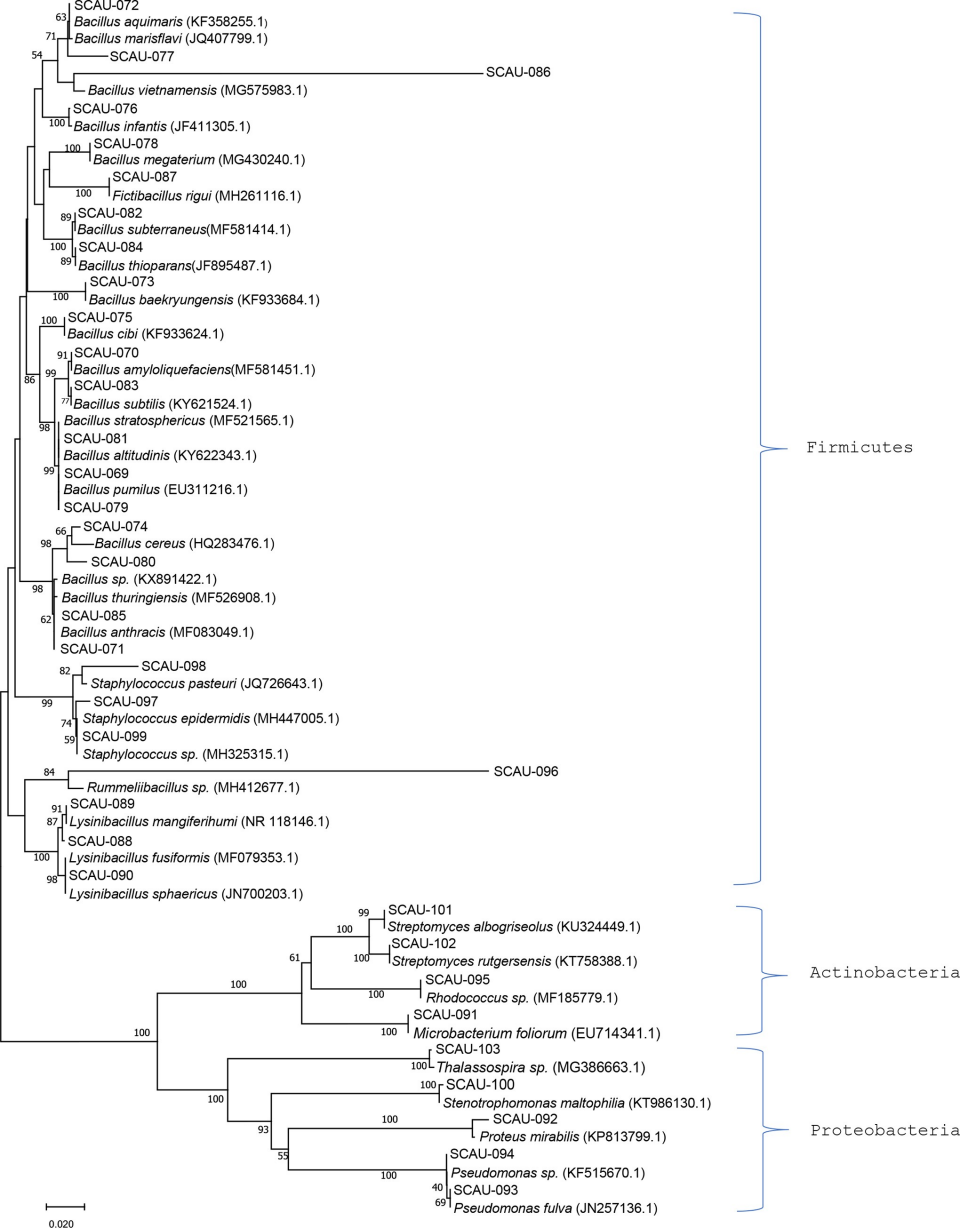
Neighbor-joining phylogenetic tree from analysis of the 16s rRNA sequences of culturable bacteria isolated from intestines of tilapia fed woody forage. The numbers at nodes were percentages indicating the levels of bootstrap support, based on a neighbor joining analysis of 1000 resampled datasets. Only values > 50% were shown. Scale bar: 0.02 substitutions per nucleotide position.

The 16S rDNA sequences of the 133 selected bacterial isolates were sequenced and compared with other sequences in GenBank. The results showed that 128 isolates shared 95–100% similarity with their closest NCBI relatives, while the five remaining isolates shared 87% similarity with their closest NCBI relatives (*Stenotrophomonas sp*. tap-10, Accession: EF221778.1). BLAST analyses showed that the 128 bacterial isolates belonged to three phyla, Firmicutes, Actinobacteria and Proteobacteria. Firmicutes accounted for 74.3% of the total isolates, which included five genera and 26 species, followed by Proteobacteria (14.3%, including four genera and five species) and Actinobacteria (11.4%, including three genera and four species). Among the 12 bacterial genera recovered in this study, *Bacillus* was the most diverse and common, with 18 species and 63 isolates identified. Further phylogenetic analysis was performed on 35 representative isolates (only one isolate was selected from all the isolates in the same species as a representative isolate), which correspondingly showed similarities to 35 different bacterial species (Fig 1). The results showed that SCAU-101, SCAU-102, SCAU-095, SCAU-091 were closely related to *Streptomyces albogriseolus*, *Streptomyces rutgersensis*, *Rhodococcus sp*. and *Microbacterium foliorum* in the phylum Actinobacteria. SCAU-103, SCAU-100, SCAU-092, SCAU-094, SCAU-093 were closely related to *Proteus mirabilis*, *Pseudomonas fulva*, *Pseudomonas sp*., *Stenotrophomonas maltophilia* and *Thalassospira sp*. in the phylum Proteobacteria. All of the remaining representative bacterial isolates belonged to the phylum Firmicutes.

### Distribution and comparison of bacteria populations among five experimental groups

**Fig 2:**
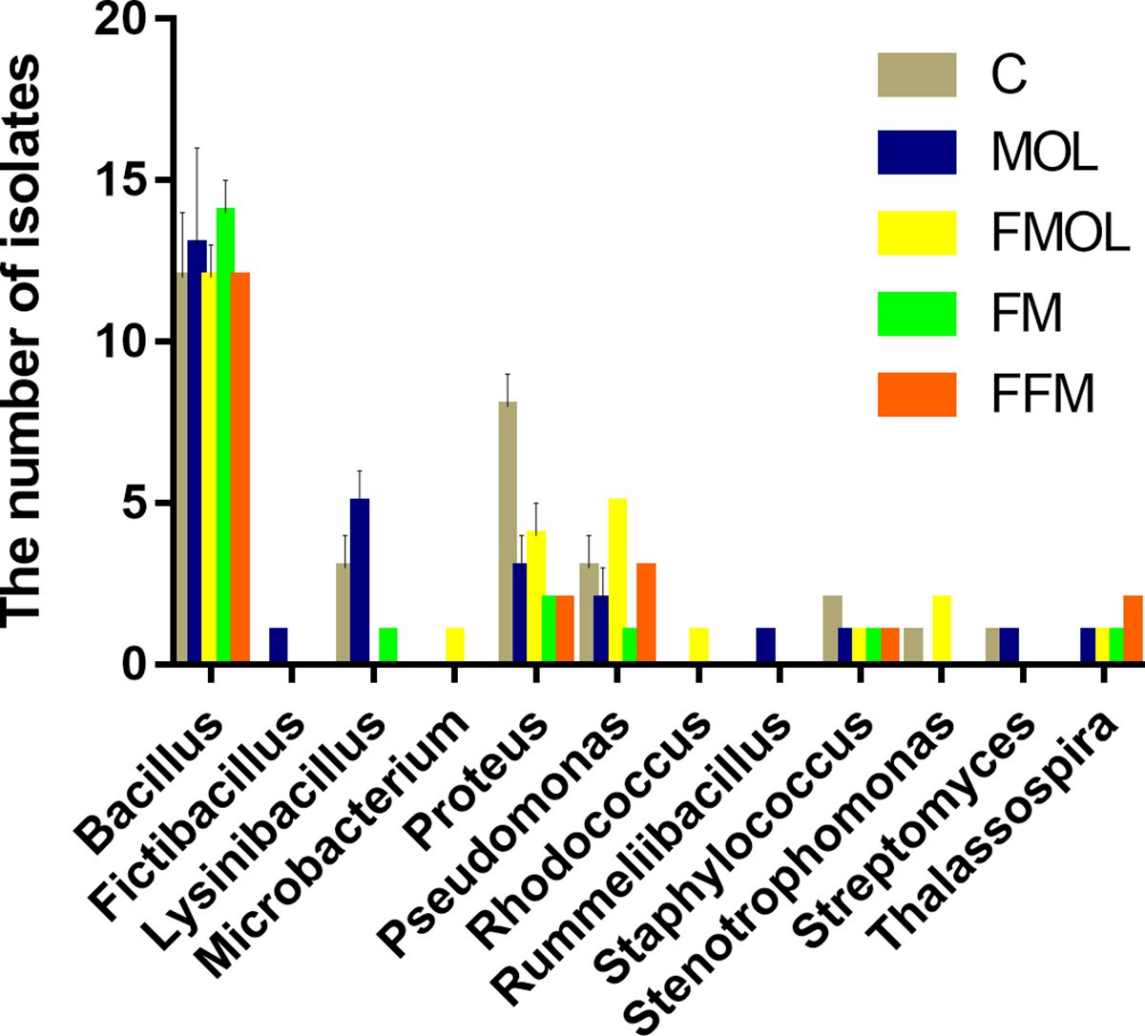
The intestinal bacteria communities at genus level. The color-coded bar plot showed the numbers of isolates of 12 genera in 5 groups.

In this study, 12 genera, including *Bacillus*, *Fictibacillus*, *Lysinibacillus*, *Microbacterium*, *Proteus*, *Pseudomonas*, *Rhodococcus*, *Rummeliibacillus*, *Staphylococcus*, *Stenotrophomonas*, *Streptomyces* and *Thalassospira* were recovered from the five tilapia groups (Fig 2). The most bacterial genera were isolated from MOL leaf meal group (nine genera), followed by fermented MOL leaf meal group (eight genera), the control group (seven genera), the FM group (six genera), and the fermented FM group (five genera). In addition, three media (LB, MRS and PIYCA) were used to recover bacteria at the same time. And, the bacteria cultured in LB medium had the highest diversity with a total of 70 culturable bacteria belonged to 28 species in 11 genera were recovered. Furthermore, 31 culturable bacteria belonging to 15 species of the 7 genera were isolated from the PIYCA medium. In addition, the MRS medium had the lowest number of culturable bacteria with 27 isolates in 11 species of 4 genera (Table 2).

**Table 2:** The situation of the identified and known bacteria in three different media.

Bacterial species (the representative isolates’ accession numbers in GenBank)	MRS	LB	PIYCA
*Bacillus altitudinis* (MK281515)	1	3	5
*Bacillus amyloliquefaciens* (MK281516)		1	
*Bacillus anthracis* (MK281517)		1	
*Bacillus aquimaris* (MK281518)		5	2
*Bacillus baekryungensis* (MK281519)	2		
*Bacillus cereus* (MK281520)		2	1
*Bacillus cibi* (MK281521)		2	
*Bacillus infantis* (MK281522)	7	2	
*Bacillus marisflavi* (MK281523)	1		
*Bacillus megaterium* (MK281524)		7	1
*Bacillus pumilus* (MK281525)	1	1	
*Bacillus sp*. (MK281526)	4	2	2
*Bacillus stratosphericus* (MK281527)		2	1
*Bacillus subterraneus* (MK281528)		2	
*Bacillus subtilis* (MK281529)	1	1	
*Bacillus thioparans* (MK281530)		3	
*Bacillus thuringiensis* (MK281531)	1		
*Bacillus vietnamensis* (MK281532)		2	
*Fictibacillus rigui* (MK281533)		1	
*Lysinibacillus fusiformis* (MK281534)			3
*Lysinibacillus mangiferihumi* (MK281535)		2	1
*Lysinibacillus sphaericus* (MK281536)			3
*Microbacterium foliorum* (MK281537)		1	
*Proteus mirabilis* (MK281538)	5	11	3
*Pseudomonas fulva* (MK281539)			1
*Pseudomonas sp*. (MK281540)	3	8	2
*Rhodococcus sp*. (MK281541)		1	
*Rummeliibacillus sp*. (MK281542)	1		
*Staphylococcus epidermidis* (MK281543)		2	
*Staphylococcus pasteuri* (MK281544)		2	
*Staphylococcus sp*. (MK281545)		1	1
*Stenotrophomonas maltophilia* (MK281546)		1	2
*Streptomyces albogriseolus* (MK281547)		1	
*Streptomyces rutgersensis* (MK281548)		1	
*Thalassospira sp*. (MK281549)		2	3
Total number of bacterial isolates	27	70	31

After further analysis of bacterial distribution, it was found that the six most abundant species were *Bacillus baekryungensis*, *Bacillus marisflavi*, *Bacillus pumilus*, *Bacillus methylotrophicus*, *Proteus mirabilis* and *Pseudomonas taiwanensis*, which were isolated from the guts of tilapia from all five experimental groups. These bacteria constitute the core flora of tilapia. Furthermore, some unique bacterial communities were present among the five feed groups. For examples, *Fictibacillus rigui*, *Lysinibacillus sphaericusare* and *Rummeliibacillu spycnus* were only isolated from the MOL leaf meal group, while *Microbacterium foliorum* and *Pseudomonas fulva* were unique to the fermented MOL leaf meal group. *Bacillus aquimaris* and *Bacillus megaterium* were recovered only from the group of fermented FM (Table 3).

**Table 3:** Bacteria in the gut in five experimental groups.

Bacterial species (the representative isolates’ accession numbers in GenBank)	C	MOL	FMOL	FM	FFM
*Bacillus altitudinis* (MK281515)		1±0.3		3±1.5	2±0.9
*Bacillus amyloliquefaciens* (MK281516)	1±0.3^b^	1±0.3			
*Bacillus anthracis* (MK281517)			1±0.3		
*Bacillus aquimaris* (MK281518)					1±0.3
*Bacillus baekryungensis* (MK281519)	2±0.3 ^b^		1±0.6	1±0.3	1±0.3
*Bacillus cereus* (MK281520)	1±0.6 ^b^				
*Bacillus cibi* (MK281521)		1±0.3	1±0.3	1±0.3	
*Bacillus infantis* (MK281522)	1±0.3 ^b^				1±0.3
*Bacillus marisflavi* (MK281523)	1±0.6 ^b^	2±0.9	1±0.6	1±0.6	
*Bacillus megaterium* (MK281524)					1±0.3
*Bacillus pumilus* (MK281525)	1±0.6 ^b^		1±0.6	2±0.9	1±0.3
*Bacillus* sp. (MK281526)		2±0.9	1±0.6	1±0.3	1±0.6
*Bacillus stratosphericus* (MK281527)		1±0.3	1±0.3		1±0.3
*Bacillus subterraneus* (MK281528)			1±0.3	1±0.3	
*Bacillus subtilis* (MK281529)		1±0.3	1±0.3		
*Bacillus thioparans* (MK281530)		1±0.3	1±0.6		
*Bacillus thuringiensis* (MK281531)	1±0.36 ^b^				
*Bacillus vietnamensis* (MK281532)		1±0.3			1±0.3
*Fictibacillus rigui* (MK281533)		1±0.3			
*Lysinibacillus fusiformis *(MK281534)	1±0.3 ^b^	1±0.3		1±0.3	
*Lysinibacillus mangiferihumi* (MK281535)	1±0.6 ^b^	1±0.3			
*Lysinibacillus sphaericus* (MK281536)		2±0.9			
*Microbacterium foliorum* (MK281537)			1±0.3		
*Proteus mirabilis* (MK281538)	4±0^a^	2±0.3	2±1.2	1±0.6	1±0
*Pseudomonas fulva* (MK281539)			1±0.3		
*Pseudomonas* sp. (MK281540)	2±0.9 ^b^	1±0.6	2±1.2	1±0.36	2±0.3
*Rhodococcus* sp. (MK281541)			1±0.3		
*Rummeliibacillu spycnus* (MK281542)		1±0.3			
*Staphylococcus epidermidis* (MK281543)	1±0.3^b^		1±0.3		
*Staphylococcus pasteuri* (MK281544)	1±0.3 ^b^			1±0.3	
*Staphylococcus* sp. (MK281545)		1±0.3			1±0.3
*Stenotrophomonas maltophilia* (MK281546)	1±0.3 ^b^		1±0.6		
*Streptomyces albogriseolus* (MK281547)		1±0.3			
*Streptomyces rutgersensis* (MK281548)	1±0.3^b^				
*Thalassospira* sp. (MK281549)		1±0.3	1±0.3	1±0.3	1±0.6

The five groups respectively are control group (C), *Moringa oleifera* Lam group (MOL), fermented *Moringa oleifera* Lam group (FMOL), *Folium mori* leaf meal group (FM) and fermented *Folium mori* leaf meal group (FFM). (The different letters a / b in the same column of the data in the table indicate that the difference is significant (P <0.05), and the letter without the shoulder indicates no significant difference in the same column).

The Bray-Curtis analysis (Table 4) showed a 62.50–87.50% dissimilarity of bacterial communities between each two groups, except for the FM and fermented FM groups, which had 100% dissimilarity, indicating that the bacterial communities between each two groups displayed obvious differences.

**Table 4:** The dissimilarity of bacteria community associated with different feed groups.

	**C**	**MOL**	**FMOL**	**FM**	**FFM**
C	/	76.50%	87.50%	63.60%	84.60%
MOL	76.50%	/	68.40%	71.40%	62.50%
FMOL	87.50%	68.40%	/	69.20%	86.70%
FM	63.60%	71.40%	69.20%	/	100%
FFM	84.60%	62.50%	86.70%	100%	/

The five groups respectively are control group (C), *Moringa oleifera Lam* group (MOL), fermented *Moringa oleifera Lam* group (FMOL), *Folium mori* leaf meal group (FM) and fermented *Folium mori* leaf meal group (FFM).

### Distribution of aerobic culturable bacteria with antibacterial activity

In this study, a total 128 isolates were belonged to 35 different known bacterial species. So, 35 representative isolates (only one isolate was selected from all the isolates in the same species as a representative isolate) were selected for antibacterial activity. All of the 35 representative isolates were tested against 4 indicator bacteria to examine their antibacterial activity against four fish pathogens including *Streptococcus agalactiae*, *Streptococcus iniae*, *Micrococcus luteus* and *Vibrio parahemolyticu*. The results (Table 5) revealed that 15 species (42.86%) displayed antibacterial activity against at least one indicator bacteria of the four pathogens. And, over 14% (5 species) of bioactive isolates displayed strong activity against the three and more indicator bacteria. Furthermore, *Bacillus amyloliquefaciens* SCAU-070 and *Streptomyces rutgersensis* SCAU-102 displayed strong activity against all four indicator bacteria, MIC values are 6.2–25 μg / ml and 3.1–25μg / ml, respectively (Table 6). Among them, the inhibitory effect of *Bacillus amyloliquefaciens* SCAU-070 on *Streptococcus*. *iniae* was the strongest, and the zone of inhibition reached 17.8 mm.

**Table 5:** Antibacterial activity of aerobic culturable bacteria isolated from tilapia gut.

Bacterial representative isolates	Diameter of the growth inhibition zone (mm)			
	SA	SI	ML	VP
*Bacillus altitudinis* (MK281515)	13.1±0.5	11.3±0.2	15.3±0.6	-
*Bacillus amyloliquefaciens* (MK281516)	15.5±0.9	17.1±0.7	13.3±0.6	12.3±0.6
*Bacillus anthracis* (MK281517)	-	-	-	-
*Bacillus aquimaris* (MK281518)	-	-	-	-
*Bacillus baekryungensis* (MK281519)	-	-	-	-
*Bacillus cereus* (MK281520)	-	-	12.3±0.4	
*Bacillus cibi* (MK281521)	-	9.3±0.3	-	-
*Bacillus infantis* (MK281522)	-	-	-	-
*Bacillus marisflavi* (MK281523)	-	-	-	-
*Bacillus megaterium* (MK281524)	-	-	-	-
*Bacillus pumilus* (MK281525)	12.4±0.9	10.2±0.4	16.2±0.5	
*Bacillus* sp. (MK281526)	-	-	-	-
*Bacillus stratosphericus* (MK281527)	-	-	11.2±0.5	-
*Bacillus subterraneus* (MK281528)	-	-		-
*Bacillus subtilis* (MK281529)	12.3±0.5	-	-	-
*Bacillus thioparans* (MK281530)	-	-	-	-
*Bacillus thuringiensis* (MK281531)	-	-	-	-
*Bacillus vietnamensis* (MK281532)	-	-	-	-
*Fictibacillus rigui* (MK281533)	-	-	9.2±0.8	-
*Lysinibacillus fusiformis *(MK281534)	-	-	-	-
*Lysinibacillus mangiferihumi* (MK281535)	9.6±0.5	12.5±0.6	-	-
*Lysinibacillus sphaericus* (MK281536)	-	-	-	-
*Microbacterium foliorum* (MK281537)	-	-	-	-
*Proteus mirabilis* (MK281538)	-	-	13.5±0.7	-
*Pseudomonas fulva* (MK281539)	-	-	-	-
*Pseudomonas* sp. (MK281540)	9.6±0.3	11.5±0.6	-	-
*Rhodococcus* sp. (MK281541)	-	-	-	-
*Rummeliibacillu spycnus* (MK281542)	-	-	-	-
*Staphylococcus epidermidis* (MK281543)	14.4±0.6	-	12.2±0.3	-
*Staphylococcus pasteuri* (MK281544)	-	-	-	-
*Staphylococcus* sp. (MK281545)	-	-	-	15.2±0.7
*Stenotrophomonas maltophilia* (MK281546)	12.5±0.7	8.4±0.7	13.2±0.3	-
*Streptomyces albogriseolus* (MK281547)	-	-	-	-
*Streptomyces rutgersensis* (MK281548)	13.5±0.8	11.4±0.6	17.2±0.2	14.9±0.8
*Thalassospira* sp. (MK281549)	-	-	-	-

SA *Streptococcus agalactiae*, SI *Streptococcus iniae*, *ML Micrococcus luteus*, *VP Vibrio Parahemolyticus*,—displayed traces or no antagonistic effects were observed

**Table 6:** The MIC value was determined by selecting strains with primary screen activity and bacteriostasis circle more than 1.5 cm. *(Bacillus altitudinis, Bacillus amyloliquefaciens, Bacillus pumilus, Staphylococcus sp., Streptomyces rutgersensis)*.

Bacterial isolates	Antibacterial activity/ MIC (μg/mL)			
	SA	SI	ML	VP
*Bacillus altitudinis* (MK281515)	12.5	25	6.2	-
*Bacillus amyloliquefaciens *(MK281516)	6.2	3.1	12.5	25
*Bacillus pumilus* (MK281525)	25	50	6.2	
*Staphylococcus* sp. (MK281545)	-	-	-	12.5
*Streptomyces rutgersensis* (MK281548)	12.5	25	3.1	12.5
Control	< 1.6	< 1.6	< 1.6	< 1.6

## Discussion

Ingestion has an important effect on the intestinal microbes and their functions in tilapia [52]. And, the microbiota can also affect behaviors [53], including feeding behavior, digestive/absorptive processes (e.g., by modulating intestinal motility and the intestinal barrier) [54], metabolism, as well as the immune response, with repercussions on the energy homeostasis, growth status [55] and health of the host. Research by Dhaneshwaree Asem et al. has shown that bacterial strains residing in the gastrointestinal region of non-ruminant swine are a promising source for lignocellulose degrading microorganisms that could be used for biomass conversion. And, few reports have investigated directly the relationships between feeding tilapia plant protein and the resulting intestinal microbes and functions. The present study investigated for the first time that the gut bacterial communities of tilapia changed greatly after feeding woody forages, including MOL, fermented MOL, FM and fermented FM. Furthermore, some unique bacterial communities were found in the guts of tilapia in this study. Our results expand the knowledge and understanding of the gut bacterial diversity of tilapia.

In this study, 133 aerobic culturable isolates were recovered and identified from the gut of tilapia from the five forage groups, with 35 species and 12 genera in three phyla (Firmicutes, Actinobacteria and Proteobacteria) identified. These results suggested that the guts of tilapia harbor abundant and diverse bacterial communities. Pakingking et al. [56] reported that 13 genera, including *Aeromonas*, *Bacillus*, *Citrobacter*, *Edwardsiella*, *Enterobacter*, *Klebsiella*, *Pasteurella*, *Photobacterium*, *Plesiomonas*, *Pseudomonas*, *Shewanella*, *Staphyloccocus* and *Vibrio* were recovered from the guts of tilapia fed artificial feed. However, among the 13 bacterial genera recovered by Pakingking et al. [56], only three genera (*Bacillus*, *Pseudomonas* and *Staphylococcus*) were identified in this study, indicating that woody feed had a significant effect on the intestinal bacterial community of tilapia. In addition, the 16S rDNA sequences of five isolates recovered in this study shared 87% similarity with their closest NCBI relatives (*Stenotrophomonas sp*. tap-10, Accession: EF221778.1), indicating that they may be new species. Further identification of the five candidate new species is necessary, including assays such as multigene analysis and morphological studies. Interestingly, Mikalsen and Colquhoun [57] isolated a *Francisella asiatica sp*. *nov*. from farmed tilapia from Costa Rica. These results suggested that the intestinal bacteria of tilapia may contain many novel bacterial species. With the rapid development of molecular biology techniques, high-throughput sequencing methods can aid in the identification of more novel microbial species from the guts of tilapia [58].

To some extent, the gut bacterial composition of tilapia from the five groups can reflect the effect of woody forage on the aerobic culturable bacterial community. For examples, the number of bacterial species and isolates recovered in the MOL and fermented MOL groups was higher than that observed in the control group. This result might have occurred because of relatively high contents of polysaccharides and trace element [59], which could be available for more bacteria to utilize. In contrast, the number and abundance of bacterial in the FM and fermented FM groups was lower than that observed in the control group. Furthermore, *Bacillus*, a common probiotic [60], accounted for the majority of the aerobic culturable bacterial community in the five groups, whereas other genera, including *Fictibacillus*, *Lysinibacillus*, *Microbacterium*, *Rhodococcus*, *Rummeliibacillus* and *Thalassospira*, were not isolated from the control group (Table 3). An interesting phenomenon was that after feeding with woody forage, the abundance and quantity of *Bacillus* in the control group were lower than those in the woody feed group. According to Table 5, *Bacillus altitudinis*, *Bacillus cibi*, *Bacillus stratosphericus*, and *Bacillus subtilis* were antagonistic to selected fish pathogens, but these four *Bacillus* species were not found in the control group. Whether woody forage affects all this requires in-depth research. These results indicated that the woody forage enriched the diversity of aerobic culturable bacteria in the guts of tilapia. In addition, conditional pathogens such as Proteus and Stenotrophomonas were rarely observed in the guts of tilapia in the FM and fermented FM groups in this study (Table 3). These results may be associated with the antibacterial activity of FM [61].

The presence of some specific bacterial species can also reflect the influences of woody forages on the diversity of aerobic culturable bacteria in the intestines. Some specific bacterial species were found in the guts of tilapia in this study. For example, *Fictibacillus rigui*, *Lysinibacillus sphaericusare* and *Rummeliibacillu spycnus* were only detected in the MOL group (Table 3). *Fictibacillus rigui* was first isolated from fresh water of the Woopo wetland in South Korea [62]. Some species of *Lysinibacillus* are positive for catalase and have broad spectrum antimicrobial activity [63]. *Rummeliibacillu spycnus* was recently shown to produce an arginine metabolic enzyme with high activity [64] and was speculated to promote better arginine absorption by tilapia on a woody diet in this study. Interestingly, *Microbacterium foliorum*, which produces a cold-active α-amylase [65], was observed in the guts of tilapia fed fermented MOL leaf meal (Table 3). The limited use of carbohydrates in the diet of fish may have something to do with the evolutionary selection of fish to live in water. In general, amylase activity in fish follows the pattern of carnivorous fish < omnivorous fish < herbivorous fish [66]. We speculated that *Microbacterium foliorum* is most likely produce some cold-active α-amylases in this special environment to promote starch digestion in the intestines. In addition, as a unique bacterial species recovered from the guts of tilapia in the fermented FM group, *Bacillus aquimaris* could also produce α-amylase [67]. It should be noted that although woody forage has certain resource advantages, it is difficult for fish to degrade the lignin and cellulose in this material. However, the bacteria in guts of fish could assist in this process. For example, *Pseudomonas fulva* was identified in the guts of tilapia in the MOL group, and *Pseudomonas* can produce ligninase and cellulase [68, 69]. In general, cellulose and lignin, as anti-nutritive factors, hinder the digestion and absorption of typical nutrient [70]. Fermenting *Pseudomonas fulva* to obtain some extracellular enzymes such as cellulase would be an excellent direction for future investigations. *Rhodococcus sp*. recovered in the guts of tilapia in the fermented MOL group may degrade steroid [71]. However, the fish guts are complex environments, and the exact functions of *Rhodococcus sp*. in guts are unknown.

As a probiotic, *Bacillus megaterium* was only isolated from the fermented FM group (Table 3). In a recent study, the application of *Bacillus megaterium* as a probiotic in feed could aid in maintaining the balance of intestinal microflora populations and increase the activity of digestive enzymes and the growth of catfish [72]. Therefore, whether *Bacillus megaterium* can also improve the growth of tilapia or even other fish is a question that is worth elucidating. In addition, *P*. *mirabilis*, a conditional pathogen [73], was also shown to be the most abundant isolate in the control group, while it was rarely observed in other woody forage groups, suggesting that the woody forage had a specific antibacterial activity [74, 75].

The present study showed that supplementation of forage modulated the adherent gut microbiota. In this respect a fundamental question arises: what effect does the gut microbiota have on the pathogenic colonization? one can hypothesize that beneficial bacteria colonizing the gut may offer protection against invading fish pathogens [76]. In vitro growth inhibition of *Streptococcus agalactiae*, *Streptococcus iniae*, *Micrococcus luteus* and *Vibrio parahemolyticus* showed that *Bacillus amyloliquefaciens* and the *Streptomyces rutgersensis* isolated in the present study have broad and efficient agonistic activities. During the last 20 years, numerous papers have suggested that *Streptomyces* used as probiotics in aquatic animals can produce bacteriocins [77], siderophores [78] and other antibacterial chemicals. The dietary supplementation of *Bacillus amyloliquefaciens* can be used to improve the health and growth rate of fish [79]. Therefore, in order to clarify whether supplementation of probiotic improves disease resistance, challenge studies have to be carried out. In addition, the remaining bacteria isolated in present study also have some antagonism to pathogenic bacteria, which was a valuable resource to be developed.

## Conclusions

In summary, in this study, the diversity and bioactivity of aerobic culturable intestinal bacteria of tilapia in five diet groups (including four woody forages and the control group) was successfully characterized. Woody forages had a great impact on the diversity of aerobic culturable bacteria in the guts of tilapia, and feeding different woody forages caused changes in the bacterial community in the gut of tilapia. In addition, a large number of bacterial resources that have antagonistic effects on pathogenic bacteria were discovered by present study. These results contribute to our knowledge and understanding of the intestinal bacterial diversity and bioactivity of tilapia and can aid in the further exploitation of new diets and probiotic source in aquaculture.
